# Comparison of Rehabilitation Outcomes Between Surgical and Conservative Treatments for Incomplete Intertrochanteric Proximal Femur Fractures—A Retrospective Cohort Study

**DOI:** 10.3390/jcm15176691

**Published:** 2026-08-28

**Authors:** Cheuk Kin Kwan, Yu Hei Choi, Rossetti Hiu Yin Lam, Richard Ku, Tsz Hang Ma, Wing Hong Liu, Wing Hoi Cheung, Chaoran Liu, Sheung Wai Law, Ronald Man Yeung Wong

**Affiliations:** 1 Department of Orthopaedics and Traumatology, The Chinese University of Hong Kong, Hong Kong SAR, China; ericckkwan@cuhk.edu.hk (C.K.K.); 1155120992@link.cuhk.edu.hk (R.H.Y.L.); 1155119894@link.cuhk.edu.hk (R.K.); 1155177046@link.cuhk.edu.hk (T.H.M.); louischeung@cuhk.edu.hk (W.H.C.); c.liu@cuhk.edu.hk (C.L.); lawsw@cuhk.edu.hk (S.W.L.); 2 Department of Orthopaedics and Traumatology, Prince of Wales Hospital, Hong Kong SAR, China; cyh478@ha.org.hk (Y.H.C.); winghongliu001@cuhk.edu.hk (W.H.L.); 3 Department of Orthopaedic Rehabilitation, Tai Po Hospital, Hong Kong SAR, China

**Keywords:** geriatric hip fracture, magnetic resonance imaging, conservative management, rehabilitation, early mobilization

## Abstract

**Background/Objectives**: Incomplete intertrochanteric proximal femur fractures pose a management dilemma regarding the choice between surgery and conservative care. This study compared radiographic and functional outcomes between surgical fixation and conservative treatment and evaluated whether immediate full weight-bearing (FWB) can be performed by conservatively managed patients. **Methods**: This is a retrospective cohort study of consecutive patients aged ≥60 who presented with MRI-confirmed incomplete intertrochanteric fractures at a tertiary trauma center and rehabilitation unit from 2010 to 2020. Clinical outcomes were compared between conservatively managed and surgically treated patients. The primary outcome was radiographic bone union at 3 months; the secondary outcomes included secondary displacement, mobility at discharge, hospital length of stay, and mortality at 3 months and 1 year. **Results**: Forty-two patients met the inclusion criteria: 10 were managed conservatively, and 32 were managed operatively. Baseline age and sex distributions were similar between groups. At 3 months post-injury, bone union rates were 100% in both groups, with no incidences of secondary displacement regardless of whether a course of protected weight-bearing was prescribed. Functional mobility at discharge and length of stay were comparable. Mortality at 3 months and 1 year did not differ significantly between groups. **Conclusions**: The preliminary findings of this retrospective cohort study demonstrated managing MRI-diagnosed incomplete intertrochanteric proximal femur fractures conservatively to be feasible. Despite the limited sample size, all included patients who were treated conservatively achieved bone union by 3 months without secondary displacement. Their clinical outcomes were also comparable to the outcomes of those who were treated with surgical fixation. However, given the small sample size and lack of randomization, further investigation is warranted before clinical application.

## 1. Introduction

Fragility-related hip fractures represent a major public health concern and are associated with substantial morbidity, mortality, and healthcare expenditure [1]. Despite successful fracture liaison services and the introduction of timely osteoporosis treatment [2,3], the total estimated number of hip fractures was also predicted to nearly double from 2018 to 2050 [4]. Hip fractures are also ranked as one of the most common disabilities and impose a growing burden on the healthcare system [5,6].

Occult hip fractures may not be obvious on routine radiography, thus requiring further imaging with advanced imaging. Magnetic resonance imaging (MRI) can more reliably detect occult hip fractures by identifying bone marrow edema, with or without obvious cortical breaks [7]. According to the Evans classification, stable intertrochanteric proximal femur fractures demonstrate features including an intact posteromedial cortex, whereas unstable patterns include comminution or reverse obliquity [8].

Early surgical fixation has long been considered the gold standard for treating intertrochanteric proximal femur fractures. However, the invasive nature of this treatment modality also carries inherent risks. Complications include surgical site infection, venous thromboembolism, bleeding, anesthetic complications, and implant-related failures [1,9]. For frail elderly patients with significant medical comorbidities, perioperative risks may render surgical treatment inappropriate, leading some physicians to consider non-surgical management despite the lack of validated selection criteria [10]. The timing of surgery is also particularly critical in hip fracture management. Surgical delays of more than 48 h have shown to increase in-patient morbidity and mortality [5].

However, for greater-trochanteric fractures with occult intertrochanteric extension, there have been emerging reports showing that conservative treatment yields satisfactory results, with 95.5% recovery of walking ability and no severe side effects [11]. Other studies found no significant differences in functional ambulation scores between surgical and non-surgical treatment groups with similar injuries [10]. Prior evidence suggests that fractures with less than 50% intertrochanteric extension can be managed non-operatively with minimal risk of secondary displacement [12]. Despite discussions in the literature, there is still a lack of consensus on whether incomplete intertrochanteric proximal femur fractures should be treated conservatively.

Another controversy lies in whether immediate full weight-bearing should be allowed in order to treat this type of injury. Many clinicians remain hesitant to permit immediate full weight-bearing (FWB) due to theoretical concerns regarding secondary displacement of the fracture under physiological loading [13]. Prolonged restricted weight-bearing in the geriatric population is strongly associated with complications, including sarcopenia, pneumonia, and thromboembolic events [14]. This study aims to evaluate whether immediate FWB protocols could be performed in conservative treatment for incomplete intertrochanteric proximal femur fractures.

## 2. Materials and Methods

A retrospective cohort study was conducted in one rehabilitation unit at a single tertiary trauma center in Hong Kong. This was a retrospective study approved by the Joint Chinese University of Hong Kong–New Territories East Cluster Clinical Research Ethics Committee (CRE Ref No.: 2018.161). The study protocol complies with the ICH-GCP and Declaration of Helsinki.

### 2.1. Inclusion and Exclusion Criteria

**Figure 1:**
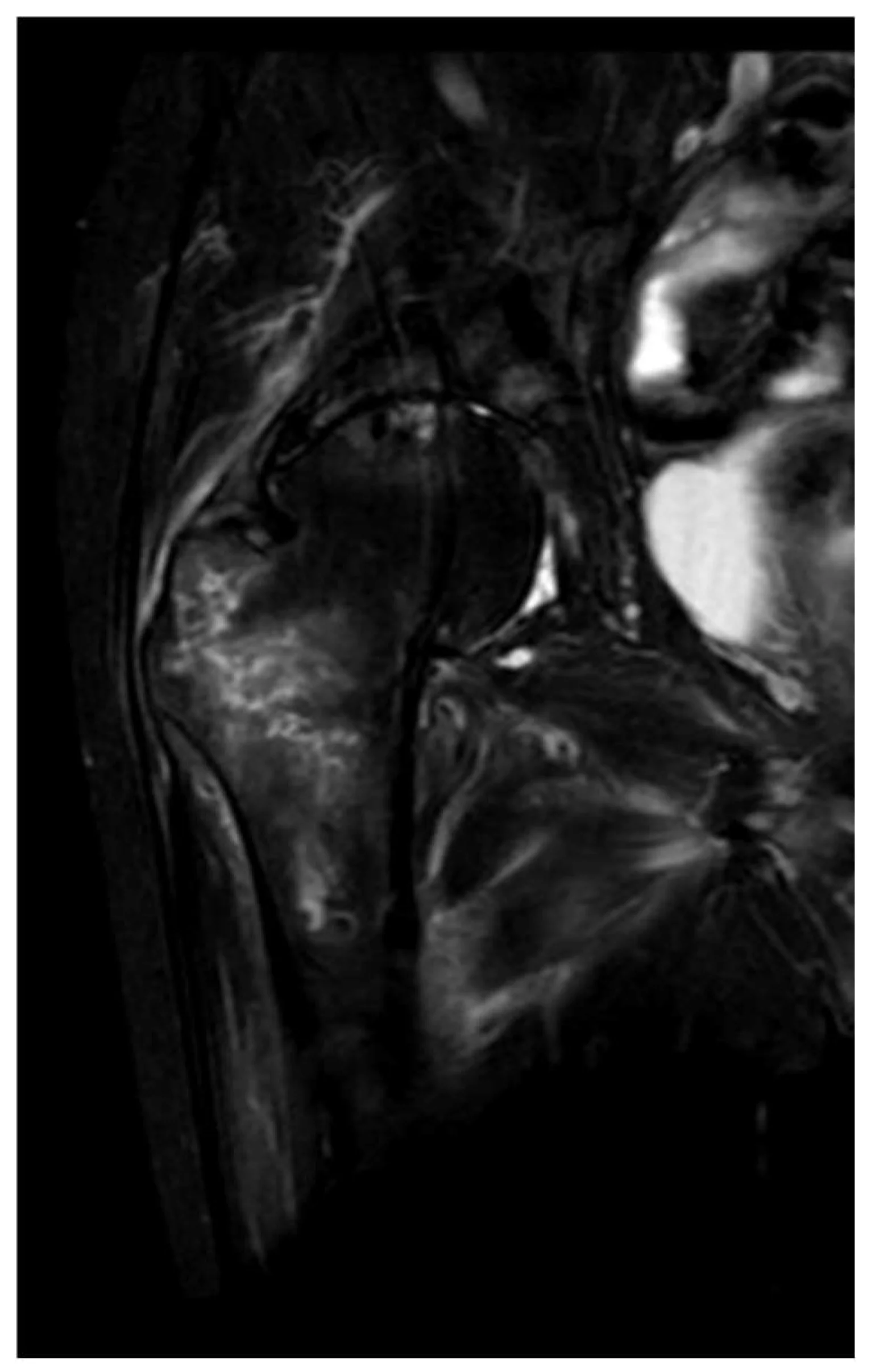
T2-weighted MRI of the right hip in coronal view showing non-displaced fracture of the greater trochanter with marrow edema involving more than 50% of the intertrochanteric region. No obvious cortical breaks of the medial calcar are present.

Consecutive patients presenting between 2010 and 2020 with incomplete intertrochanteric proximal femur fractures were identified in the hospital’s trauma database. All patients included in this analysis met the following criteria: (1) The patients had to be geriatric (aged 60 years old or above). (2) The patients had to have MRI-diagnosed incomplete intertrochanteric proximal femur fractures, defined with a T2-weighted coronal plane series. Cuts showing both the greater and lesser trochanter were chosen and assessed regarding the percentage of marrow edema extending between these two landmarks (Figure 1). Patients with marrow edema involving more than 50% of this intertrochanteric region, without any obvious cortical breaks of the medial calcar, were selected. When multiple cuts demonstrated intertrochanteric marrow edema, the cut showing the highest percentage of involvement was chosen. Images were reviewed by a specialist in orthopedics and traumatology with >=6 years of experience in managing geriatric hip fractures. (3) Patients had to have been managed conservatively. Patients who experienced secondary fractures or polytrauma were excluded. For comparison, another cohort of geriatric patients with a similar injury but who were managed surgically with a cephalomedullary device was conveniently sampled within the study period.

In this study, conservative management was defined as early mobilization with or without immobilization with a spica brace, physiotherapy, or pain management without surgical fixation. The indication and duration of protected weight-bearing and the permitted proportion of bodyweight allowed on the injured limb were based on the clinical decision made at the time, with no published consensus. Pain control was optimized by multimodal analgesic regimens provided by the pain team. Walking exercises within the permitted weight-bearing limit were performed under the supervision of trained physiotherapists at least once a day. In the institution where this study was performed, incomplete intertrochanteric proximal femur fractures with marrow edema affecting more than 50% of the intertrochanteric region are usually treated with a cephalomedullary device, with full weight-bearing allowed afterwards. Conservative treatment was only considered upon refusal from the patient or when the patient was unfit for surgery.

### 2.2. Outcome Measures

The primary outcome was radiographic bone union at 3 months post-injury assessed by orthopedic surgeons. Fracture union was defined as the presence of radiographic healing in follow-up radiographs, characterized by the formation of a bridging callus or trabeculae, together with clinical resolution of localized pain. Fracture union is deemed complete when both clinical and radiographic criteria are met. Secondary outcomes include secondary displacement, defined by a fracture gap or step-off of more than 4 mm or a change in neck–shaft angle of more than 10 degrees; hospital length of stay, from admission to acute hospital care and discharge from rehabilitation hospital; mortality at 3 months and 1 year post-injury; and mobility at discharge, defined using the modified functional ambulatory categories (MFACs). The MFAC system is a tool dividing mobility into 7 categories, ranging from bedbound patients in category 1 to independent outdoor walkers in category 7 [15]. The detailed breakdown and descriptions are described in Table 1.

### 2.3. Data Collection

Baseline demographic and clinical data were extracted from electronic medical records, including age, sex, time from admission to surgery, and rehabilitation plan. MRI films were reviewed to confirm fracture characteristics. Radiographs obtained on admission, immediately post-operation, and 3 months post-operation were reviewed for outcome assessment.

The weight-bearing protocols were defined as follows: immediate full weight-bearing (FWB) was defined as mobilization as tolerated without any weight-bearing restrictions starting from Day 1 of admission, encouraging early gait training with a walking aid as needed. All other rehabilitation plans, ranging from partial weight-bearing to complete bedrest, were classified into the protected weight-bear (PWB) group.

### 2.4. Statistical Analysis

Continuous variables were reported as means with standard deviations if appropriate. Categorical variables were reported as frequencies and percentages if appropriate. Means of continuous variables were compared with an independent *t*-test. Ordinal data were compared using the Mann–Whitney U test. Differences in categorical variables were compared with the Chi-squared test or with Fisher’s exact test in case of inadequate cell counts. A two-tailed *p*-value < 0.05 was considered statistically significant.

## 3. Results

### MRI-Diagnosed Incomplete Trochanteric Fractures

A total of 42 patients with MRI-diagnosed incomplete intertrochanteric proximal femur fractures with more than 50% intertrochanteric extension and no cortical breakage of the medial calcar were included in this study. Of these patients, 10 of them were managed conservatively (23%), whilst 32 (77%) of them underwent surgical fixation with a cephalomedullary device (Gamma3 Nailing System, 170–180 mm, Stryker). All surgically treated patients were allowed to engage in immediate full weight-bearing with the limb operated on. The baseline characteristics of the two groups were comparable. The average age was 86.1 (SD = 8.2) years old for the conservative group and 87.2 (SD = 7.8) in the surgical group, and the difference was not statistically significant (*p* = 0.74). The gender distribution was also similar between the groups, with 60% female predominance in the conservative group and 65.625% in the group operated on (*p* = 0.88) (Table 2).

Except for patients who passed away before the 3-month follow-up, all patients received a radiographic assessment of the fracture at 3 months and were found to have achieved radiographic bone union with no signs of secondary displacement. Amongst the conservatively managed patients, 40% received a course of protection with a hip spica brace for the injured limb and engaged in protected weight-bearing. The remaining 60% engaged in immediate full weight-bearing (FWB) with no hip spica. The decision on whether to prescribe a course of protection was based on the clinical decision at the time, with no definite published consensus. All surgically managed patients were allowed to engage in immediate FWB with the treated limb.

The average duration of hospital stay was 31.6 (SD = 16.4) days in the conservative group, which can be compared to 31.1 (SD = 11.9) days in the surgical group. The difference is not statistically significant (*p* = 0.94).

Mobility at hospital discharge was comparable between the two groups (*p* = 0.83).

The 3-month mortality rate was higher in the conservative group when compared to the surgical group (10.0% vs. 3.1%); however, the difference was not statistically significant (*p* = 0.36).

Rehabilitation and clinical outcomes are summarized in Table 3.

## 4. Discussion

### 4.1. Potential of Non-Operative Treatment Regarding Incomplete Intertrochanteric Proximal Femur Fractures

In this retrospective cohort study of 42 patients with MRI-diagnosed incomplete intertrochanteric proximal femur fractures with more than 50% intertrochanteric extension, all fractures in both the conservative and surgical groups achieved radiographic bone union by 3 months, in the absence of secondary displacement.

Compared to surgical fixation via cephalomedullary devices, the patients who were conservatively managed did not exhibit significant differences in mortality rate, hospital length of stay, or mobility status at discharge (*p* > 0.05 for all groups). The Charlson comorbidity index (CCI) indicated that there was a greater comorbidity burden in the conservatively managed group than in the surgically managed group, raising concerns regarding baseline imbalance between the cohorts. Nevertheless, despite poorer baseline health, the patients managed conservatively achieved rehabilitation outcomes comparable to those of surgically managed patients.

Our findings are consistent with several studies focusing on the management of incomplete introchanteric proximal femur fractures. Both Alvarez et al. [11] and Kent et al. [16] highlighted that isolated greater-trochanteric fractures, including those with intertrochanteric extension on MRI, could still achieve satisfactory clinical outcomes following conservative management. Another more recent retrospective case series published in 2025 reviewed the outcomes of 45 incomplete, MRI-diagnosed intertrochanteric hip fractures treated conservatively, with immediate full weight-bearing walking allowed. None of the included patients experienced secondary displacement, supporting conservative treatment with immediate full weight-bearing for incomplete intertrochanteric hip fractures [17]. A similar retrospective study published in 2023 compared the clinical outcomes between surgical and conservatively managed greater-trochanter fractures with occult intertrochanteric extensions, again supporting the possibility of conservative management in this selected population [10]. The current study further strengthens the evidence by demonstrating the same observation in a cohort of older Asian adults. We also restricted the analysis to fractures with intertrochanteric extension of 50% or more, which were routinely managed surgically at our institution.

### 4.2. Potential of Immediate Full Weight-Bearing

Six (60%) patients with MRI-diagnosed incomplete intertrochanteric proximal femur fractures were permitted to engage in immediate full weight-bearing. All these patients achieved bone reunion by 3 months, with no signs of secondary displacement. Historically, the hesitation to allow immediate FWB has been driven by a perceived risk of fragment displacement or mechanical failure [13]. Despite the limited sample size, our data suggests that immediate full weight-bearing may be suitable for carefully selected incomplete intertrochanteric proximal femur fractures. Theoretically, immediate FWB offers clinical benefits by mitigating complications such as deep-vein thrombosis and pulmonary infections, which are primary drivers of morbidity in geriatric patients [14,18,19].

### 4.3. Limitations

There was a limited sample size of MRI-diagnosed incomplete intertrochanteric proximal femur fractures. With only 10 patients in the conservative group, the study had limited power with which to detect modest but clinically meaningful differences between groups. Additionally, only six patients in the MRI-defined conservative cohort were permitted to engage in immediate full weight-bearing. The risk of secondary displacement cannot be excluded despite the absence of observed events.

Selection bias was also a significant concern, and the reasons include the following: First, treatment assignment was not randomized but based on patient preferences. Our center offers operations for incomplete intertrochanteric proximal femur fractures with marrow edema involving more than 50% of the intertrochanteric region, and we collected conservatively managed cases when patients refused or were unfit for surgery. Without randomization, we could not exclude the possibility that differences in unmeasured confounders had an impact. In fact, the Charlson comorbidity index (CCI) value was greater in the conservatively managed group in the current study. Nevertheless, these patients achieved rehabilitation outcomes comparable to those of surgically managed patients despite their poorer baseline health. Consideration of conservative management for carefully selected patients with incomplete intertrochanteric fractures of the proximal femur remains valid. Another limitation of the current study is that there is currently no standardized classification for incomplete or occult intertrochanteric hip fractures. This raises the concern that the included population may not be reliably compared with populations in other published studies.

### 4.4. Future Directions

This study is more hypothesis-generating than confirmatory due to the small sample size and non-randomized design. Therefore, a randomized controlled trial (RCT) on conservative management with MRI-diagnosed incomplete trochanteric fractures should be conducted on a larger sample to validate our observations. We propose that this RCT should feature standardized MRI criteria for inclusion, a clear definition of intertrochanteric extension percentage, a standardized conservative and surgical protocol, blinding of outcome assessors, a longer follow-up period of at least 1–2 years, and inclusion of additional outcome measures such as return to pre-injury function, fall recurrence, and quality of life.

## 5. Conclusions

The preliminary findings of this retrospective cohort study demonstrate the possibility of managing MRI-diagnosed incomplete intertrochanteric proximal femur fractures conservatively. Despite the limited sample size, all included patients who were treated conservatively achieved bone union by 3 months without secondary displacement. Their clinical outcomes were also comparable to those who were treated with surgical fixation. However, given the small sample size and lack of randomization, further investigation is warranted before clinical application.

## Figures and Tables

**Table 1:** Modified functional ambulatory category (MFAC).

Category	Brief Description	Definition
1	Bedbound	Patient is unable to sit for 1 min without back and handle support
2	Sitter	Patient is able to sit without back and hand for support but requires help from two or more people to walk
3	Dependent walker	Patient requires continuous support from one person to walk
4	Assisted walker	Patient requires intermittent support from one person to walk
5	Supervised walker	Patient requires verbal supervision or standby help from one person without physical contact
6	Indoor walker	Patient can walk independently on level ground but requires help on stairs, slopes, or uneven surfaces
7	Outdoor walker	Patient can walk independently anywhere

**Table 2:** Baseline demographic characteristics of MRI-diagnosed incomplete trochanteric fractures.

Characteristics	Conservative (*n* = 10)	Operated (*n* = 32)	*p*-Value
Sample size	10	32	-
Age, mean (SD) (years)	86.1 ± 8.2	87.2 ± 7.8	0.74
Female ratio (number of female patients)	60% (6)	65.625% (11)	0.88
Charlson comorbidity index	7.5 ± 1.9	6.0 ± 1.4	0.01
Mean time from admission to surgery (days)	-	2.7 ± 1.3	-

SD: Standard deviation.

**Table 3:** Rehabilitation outcomes regarding MRI-diagnosed incomplete trochanteric fractures.

Outcome	Conservative (*n* = 10)	Operation (*n* = 32)	*p*-Value
Protected weight-bearing	40% (4/10)	0% (0/32)	-
Bone union at 3 months post-injury (excluding patients who passed away within 3 months)	100% (9/9)	100% (31/31)	-
Secondary displacement rate	0%	0%	-
MFAC at discharge, median (range)	4 (2–6)	3 (1–6)	0.83
Hospital length of stay * (SD) (days)	31.6 ± 16.4	31.1 ± 11.9	0.94
3-month mortality rate (number of patients)	10.0% (1)	3.1% (1)	0.42
1-year mortality (number of patients)	10.0% (1)	12.5% (4)	-

SD: standard deviation; MFAC: modified functional ambulation category. * Hospital length of stay, from admission to acute hospital care and discharge from rehabilitation hospital.

## Data Availability

Data are contained within the article. Individual data are unavailable due to privacy or ethical restrictions.

## Abbreviations

The following abbreviations are used in this manuscript:

MRI	Magnetic Resonance Imaging
FWB	Full Weight-Bearing
PWB	Protected Weight-Bearing
SD	Standard Deviation
MFAC	Modified Functional Ambulation Category
RCT	Randomized Controlled Trial
